# Melatonin potentiates the antineoplastic effects of venetoclax in acute myeloid leukemia cells

**DOI:** 10.1016/j.htct.2026.106534

**Published:** 2026-09-20

**Authors:** Vahid Vahedian, Keli Lima, Bruna Oliveira de Almeida, Rita de Cássia Cavaglieri, João Agostinho Machado-Neto, Eduardo Magalhães Rego

**Affiliations:** a Laboratory of Medical Investigation in Pathogenesis and Targeted Therapy in Onco-Immuno-Hematology (LIM-31), Department of Clinical Medicine, Division of Hematology and Hemotherapy, Faculty of Medicine, University of São Paulo, São Paulo, Brazil; b DO’r Institute for Education and Research (IDOR), Sao Paulo, Brazil; c Department of Pharmacology, Institute of Biomedical Sciences, University of São Paulo (USP), São Paulo, Brazil

Dear Editor,

Acute myeloid leukemia (AML) is an aggressive and biologically heterogeneous hematologic malignancy characterized by the clonal expansion of immature myeloid precursors driven by diverse cytogenetic and molecular alterations [1]. The disease predominantly affects older adults and remains associated with poor clinical outcomes, particularly in patients who are unfit for intensive chemotherapy due to comorbidities and adverse-risk disease biology [2]. Despite standard myelosuppressive chemotherapy approaches, including anthracycline-cytarabine-based induction and consolidation with or without allogeneic hematopoietic stem cell transplantation, relapse rates remain high, largely due to the persistence of therapy-resistant leukemic stem cells or clonal evolution or both [3].

Advances in the understanding of AML biology have enabled the development of targeted therapies, among which venetoclax, a selective BCL2 inhibitor, has emerged as a transformative agent [2]. As a BH3 mimetic, venetoclax restores apoptotic signaling by inhibiting the antiapoptotic protein BCL2, promoting mitochondrial outer membrane permeabilization and caspase-dependent cell death [1]. Clinically, venetoclax-based combinations, particularly with hypomethylating agents, have significantly improved response rates and survival in elderly or unfit AML patients, establishing a new therapeutic standard of care [3]. However, long-term remission is infrequent and responses are strongly influenced by molecular subtypes and resistance mechanisms, underscoring the need for optimized combinatorial and precision-based strategies [2].

Melatonin (N-acetyl-5-methoxytryptamine), a pineal-derived indoleamine, has emerged as a potent oncostatic and immunomodulatory molecule with antileukemic properties. Evidence indicates that melatonin exerts antiproliferative and proapoptotic effects on leukemia cells through the modulation of multiple intracellular signaling pathways [4]. Notably, melatonin targets several hallmarks of cancer, including uncontrolled cell proliferation, resistance to cell death, and the ability to metastasize, through cytostatic and proapoptotic mechanisms [5]. In human myeloid leukemia models, such as HL-60 cells, melatonin inhibited proliferation and induced apoptosis via mitochondrial-dependent mechanisms involving cytochrome C release and caspase activation [6]. Mechanistically, melatonin regulates key apoptotic mediators, including BCL2 family proteins, reactive oxygen species (ROS), and mitochondrial membrane permeability, thereby promoting intrinsic apoptotic signaling [4].

Additionally, melatonin suppresses leukemogenic pathways such as RBFOX3/hTERT and NF-κB/COX2 signaling, reducing tumor cell proliferation and enhancing apoptosis [7]. Importantly, it enhances the proapoptotic activity of the BH3 mimetic ABT-737 in THP-1 acute monocytic leukemia cells, supporting its role in sensitizing leukemic cells to BCL2-targeted therapies through amplification of mitochondrial apoptotic signaling [8]. Furthermore, melatonin does not interfere with the cytotoxic effects of conventional chemotherapeutic agents such as cytarabine, daunorubicin, and etoposide and may, in some contexts, enhance drug-induced cytotoxicity, highlighting its safety and potential as an adjuvant agent [9]. Collectively, these findings support melatonin as a promising adjuvant capable of potentiating therapeutic efficacy, particularly in combination with BCL2-targeted strategies.

In the present study, we investigated the effects of melatonin alone or in combination with venetoclax in AML models exhibiting intrinsic resistance to venetoclax [10]. MOLM-13 (venetoclax-sensitive), Kasumi-1 (intermediate resistance), and U-937 and OCI-AML3 (high resistance) cell lines were used. Cells were maintained in RPMI-1640 or α-MEM supplemented with 10% fetal bovine serum (FBS) and 1% penicillin/streptomycin at 37°C in a humidified atmosphere with 5% CO_2_, in accordance with American Type Culture Collection (ATCC) and Deutsche Sammlung von Mikroorganismen und Zellkulturen (DSMZ) guidelines. Cell viability was assessed using the 3-(4,5-dimethylthiazol-2-yl)-2,5-diphenyltetrazolium bromide (MTT) assay (Sigma-Aldrich, St. Louis, MO, USA) following treatment with increasing concentrations of melatonin (Sigma-Aldrich) and/or venetoclax (TargetMol, Boston, MA, USA; Figure 1A), as previously described [10]. The half maximal inhibitory concentration (IC_50_) values were determined by nonlinear regression using GraphPad Prism 8 (GraphPad Software, Inc., San Diego, CA, USA). Drug interaction effects were evaluated using ZIP synergy scores calculated with SynergyFinder (https://synergyfinder.fimm.fi/). Statistical significance was defined as p <0.05. Western blot analysis was performed to assess total and cleaved PARP1 (#56197), γH2AX (#9718), BCL2 (#15071), BCL-XL (#2764), MCL1 (#5453), p-ERK1/2 (#9101), total ERK1/2 (#9102), p-S6RP (#4858), and total S6RP (#2217), using α-tubulin (#2144) or GAPDH (#5174) as loading controls (Cell Signaling Technology, Danvers, MA, USA).

**Figure 1:**
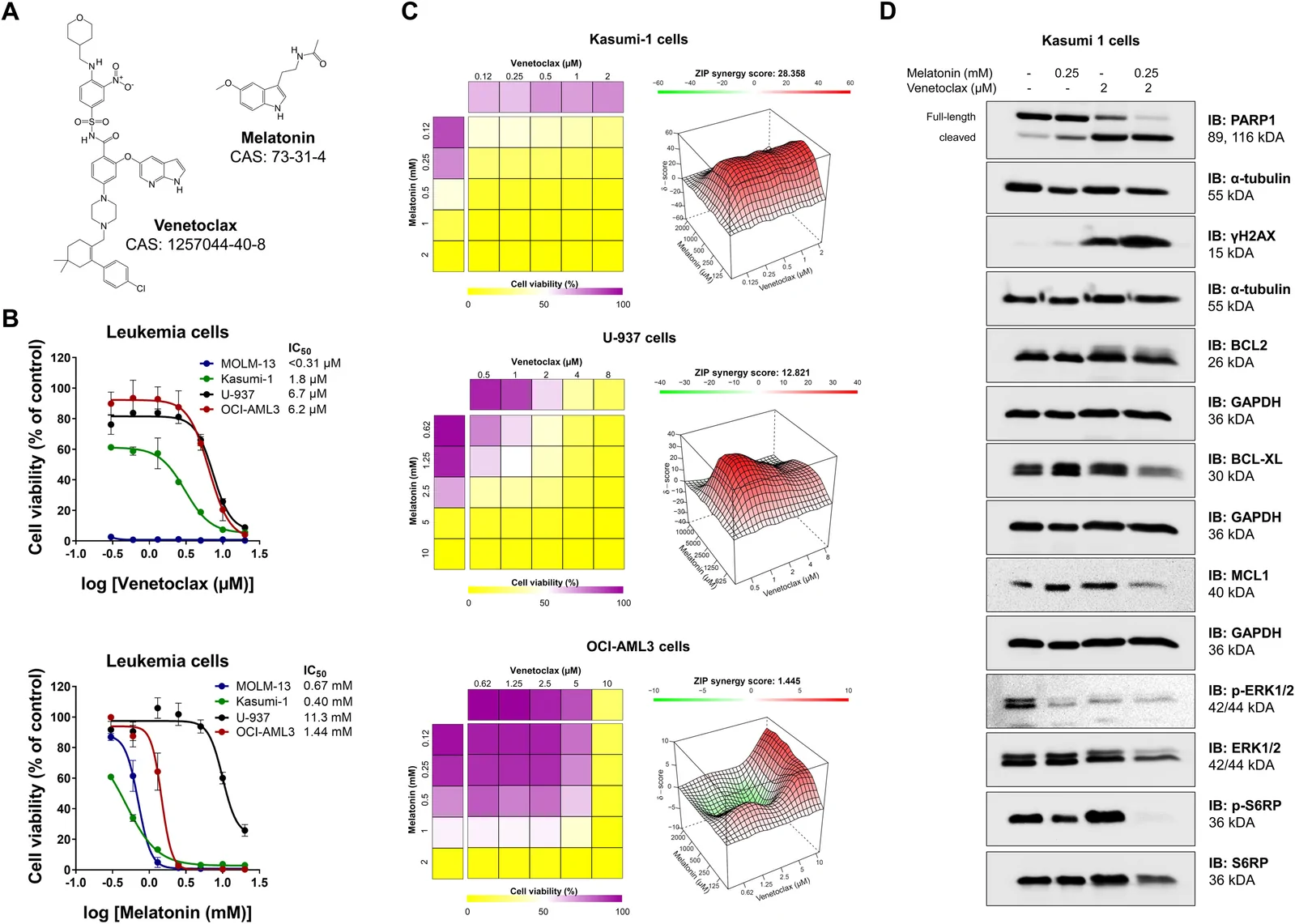
Melatonin enhances venetoclax-induced cytotoxicity in AML cell lines. (A) Chemical structures of venetoclax (CAS: 1257044-40-8) and melatonin (CAS: 73-31-4). (B) Concentration–response cytotoxicity was evaluated using an MTT (3-(4,5-dimethylthiazol-2-yl)-2,5-diphenyltetrazolium bromide) tetrazolium assay in a panel of acute myeloid leukemia cell lines treated with vehicle or increasing concentrations of venetoclax or melatonin for 72 h. Values are expressed as the percentage of viable cells relative to vehicle-treated controls. IC_50_ values and corresponding cell lines are indicated in the Figure. (C) Concentration-response cytotoxicity of combination treatments was assessed by MTT assay in Kasumi-1, U-937, and OCI-AML3 cells treated with increasing concentrations of venetoclax and melatonin, either alone or in combination, for 72 h. Cell viability is expressed as a percentage relative to vehicle-treated controls. Results represent the mean of at least three independent experiments. (D) Western blot analysis of total and cleaved PARP1, γH2AX, BCL2, BCL-XL, MCL1, phosphorylated ERK1/2 (p-ERK1/2), total ERK1/2, phosphorylated S6RP (p-S6RP), and total S6RP in whole-cell extracts from Kasumi-1 cells treated with vehicle, venetoclax, and/or melatonin at the indicated concentrations for 72 hours. Membranes were reprobed for α-tubulin or GAPDH as loading controls and developed using SuperSignal™ West Dura Extended Duration Substrate with a G:BOX Chemi XX6 imaging system.

We first assessed the degree of venetoclax resistance across the selected AML models (Figure 1B). We then evaluated the effects of melatonin and observed that MOLM-13 and Kasumi-1 cells were the most sensitive (IC_50_ = 0.67 and 0.40 mM, respectively), OCI-AML3 cells displayed intermediate sensitivity (IC_50_ = 1.44 mM), and U-937 cells were the most resistant (IC_50_ = 11.3 mM). Combination analysis revealed synergistic effects in Kasumi-1 and U-937 cells (ZIP scores of 28.35 and 12.82, respectively), whereas an additive effect was observed in OCI-AML3 cells (ZIP score = 1.44) (Figure 1C). Based on these findings, we further investigated the molecular effects in Kasumi-1 cells and found that the combination of venetoclax and melatonin markedly increased apoptotic and DNA damage markers (cleaved PARP1 and γH2AX, respectively) while reducing key proteins associated with venetoclax resistance [10,11], including BCL-XL and MCL1 expressions, and activation of the PI3K/AKT/mTOR and MAPK pathways (Figure 1D).

This study provides mechanistic insight into the synergistic antileukemic activity of melatonin combined with venetoclax, supporting a rational strategy to enhance apoptosis in AML. Venetoclax induces apoptosis through selective inhibition of BCL2; however, resistance frequently arises through compensatory upregulation of alternative antiapoptotic proteins, particularly MCL1 and BCL-XL, which sustain leukemic cell survival [3]. In this context, our findings demonstrated downregulation of both MCL1 and BCL-XL under combination treatment, providing evidence for overcoming key resistance mechanisms.

Melatonin exerts pleiotropic antineoplastic effects by modulating multiple signaling pathways, including inhibition of PI3K/AKT/mTOR, NF-κB, and ERK pathways, and activation of proapoptotic mediators such as p53 and caspases [12,13]. It also directly regulates mitochondrial apoptosis by shifting the balance between proapoptotic (BAX, BAK) and antiapoptotic (BCL2, BCL-XL, MCL1) proteins, promoting cytochrome c release and apoptosome formation [4]. Additionally, melatonin enhances the efficacy of chemotherapeutic agents and can overcome multidrug resistance through modulation of redox homeostasis and apoptotic signaling networks [14].

Emerging evidence further highlights melatonin as a time-dependent oncological adjunct that modulates tumor biology through circadian regulation, redox balance, and enhanced therapeutic responsiveness. Current data suggest that melatonin is not merely a sleep-regulating hormone, but a biologically active molecule at the intersection of circadian biology and oncology [15]. This circadian-linked modulation may further potentiate venetoclax activity by synchronizing apoptotic signaling and increasing cellular susceptibility to mitochondrial disruption.

Altogether, our findings support a model in which melatonin acts as a multifunctional adjuvant that potentiates venetoclax efficacy by targeting complementary apoptotic and resistance pathways. The observed reduction in key resistance-associated proteins (MCL1 and BCL-XL) is consistent with previous reports demonstrating that melatonin downregulates survival pathways and sensitizes tumor cells to apoptosis-inducing agents. This combinatorial approach represents a promising and innovative therapeutic strategy in AML, particularly for overcoming venetoclax resistance and improving clinical outcomes through the precision-based modulation of mitochondrial apoptosis.

## Data availability

Data will be made available on request.

## Conflicts of interest

The authors declare no competing interests.
